# Experiment in semi-natural conditions did not confirm the influence of malaria infection on bird attractiveness to mosquitoes

**DOI:** 10.1186/s13071-022-05292-w

**Published:** 2022-06-02

**Authors:** Camille-Sophie Cozzarolo, Romain Pigeault, Julie Isaïa, Jérôme Wassef, Molly Baur, Olivier Glaizot, Philippe Christe

**Affiliations:** 1 Department of Ecology and Evolution, University of Lausanne, Lausanne, 1015 Switzerland; 2 Laboratoire EBI, Equipe EES, UMR CNRS 7267, University of Poitiers, Poitiers, 86000 France; 3 Musée Cantonal de Zoologie, Lausanne, 1014 Switzerland; 4 Biogéosciences, UMR 6282, CNRS, Université Bourgogne Franche-Comté, 6 boulevard Gabriel, 21000 Dijon, France

**Keywords:** Attraction, *Culex pipiens*, Exposure, Extended phenotype, Manipulation, Parasite, *Plasmodium*, Vector

## Abstract

**Background:**

Changes in host phenotype following parasite infection are often considered as host manipulation when they seem advantageous for the parasite. However, putative cases of host manipulation by parasites are rarely tested in field-realistic conditions. Infection-induced phenotypic change cannot be conclusively considered as host manipulation if no evidence shows that this trait is adaptive for the parasite in the wild. *Plasmodium* sp., the parasites causing malaria in vertebrates, are hypothesized to “manipulate” their host by making their odour more attractive to mosquitoes, their vector and final host. While this is fairly well supported by studies on mice and humans, studies focusing on avian malaria give contradictory results.

**Methods:**

In the present study, genotyped birds at different stages (uninfected, acute and chronic) of *Plasmodium relictum* infection were exposed, in a large outdoor aviary, to their natural vector, the mosquito *Culex pipiens*.

**Results:**

After genotyping the blood meals of more than 650 mosquitoes, we found that mosquitoes did not bite infected birds more than they bit them before infection, nor more than they bit uninfected hosts.

**Conclusions:**

Our study highlights the importance of testing ecological behaviours under natural conditions and suggests that different processes might be at play in mammals and birds regarding potential manipulation of attractiveness by malaria parasites.

**Graphical Abstract:**

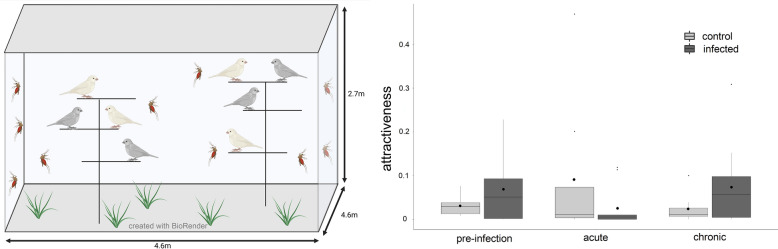

**Supplementary Information:**

The online version contains supplementary material available at 10.1186/s13071-022-05292-w.

## Background

Various groups of parasites induce alterations of their host’s behaviour [1, 2], morphology [3] and/or physiology [4, 5] in ways that increase their own fitness. Although rarely empirically demonstrated to be an expression of parasites’ genotype [6], modification of host phenotypes after parasite infection is often assumed to be host manipulation. This hypothesis has been investigated for a wide range of host/parasite associations [7], and for heteroxenous parasites in particular, where manipulation of one of the hosts (intermediate or definitive) would promote transmission of the parasite to the subsequent host [8]. One of the most widely studied and discussed cases to date is that of the malaria agent, *Plasmodium* [8].

*Plasmodium* is thought to influence the feeding behaviour of infected vectors (i.e. mosquitoes) in ways that could increase transmission to the vertebrate host. For instance, mosquitoes infected with sporozoites—the *Plasmodium* transmissible stage, present in mosquito salivary glands—showed longer probing time [9] and bit longer [10] and more frequently on multiple hosts [11] than uninfected vectors did. These changes could be general consequences of mosquitoes’ immune response and not specifically *Plasmodium* extended phenotype, as was demonstrated for infection stage-specific changes in host-seeking behaviour [12]. Another hypothesis is that vertebrate host attractiveness to vectors is increased by the presence of *Plasmodium* parasites in their blood (e.g. [13, 14]). Host-seeking mosquitoes rely on volatile molecules emitted by vertebrates to detect and select their blood source. Mice and humans infected with *Plasmodium chabaudi* and *Plasmodium falciparum*, respectively, produce some molecules in higher quantities (reviewed in [15, 16]). Some of them were shown to attract more mosquitoes when added to the smell of a healthy host [16, 17]. No such molecule has yet been identified in birds [18].

*Plasmodium*-induced alteration of host attractiveness is supported by several lab experiments in mammals [13, 17, 19–22] and birds [14, 18, 23]. However, a non-negligible number of studies showed an avoidance of infected hosts [24–27] or no difference in attractiveness [28]. These contrasting results may be explained by methodological differences, such as the possibility of host defensive behaviour during the experiment or whether infected hosts with or without transmissible stages were analysed as distinct categories [29]. The temporal dynamics of *Plasmodium* infection within a vertebrate host could also be another confusing factor. Blood stages of malaria infection are made up of an acute phase, a few days of high parasitaemia (infection intensity), followed by a chronic phase, with low parasitaemia. A previous study highlighted variations in mosquito host choice throughout the course of the infection. While no mosquito preference was detected during the acute phase of infection, vectors showed a preference for infected hosts during the chronic phase [14]. Among the hypotheses proposed by the authors, it is suggested that this temporal variation in mosquito host choice may be the result of two antagonistic effects: the attraction induced by the manipulating parasite and the repellence induced by an altered blood meal [14]. Indeed, blood quality for the mosquito, in particular its nutritional value, might vary with parasitaemia, as the number of red blood cells decreases due to their destruction at the end of the erythrocytic cycle of parasite replication [30, 31]. In addition, while the conditions making malaria infection costly for mosquito fitness are still unclear [32–35], the ingestion itself of highly parasitized blood could have a negative impact on mosquito survival ([36] but see [31]).

Most of the studies assessing the impact of host infection status on vector attractiveness have been carried out under laboratory conditions. Yet, the adaptive value of a potentially manipulable trait needs to be assessed under conditions that are "as natural as possible" [6]. While methods used in lab experiments provide a measure of pure difference in attraction, they do not allow us to determine whether this difference is field-relevant. For example, infection may alter hosts’ behaviour in ways that make them more or less easily bitten by mosquitoes [19, 37].

In the present study, we compared the number of mosquito bites received by uninfected and *Plasmodium*-infected birds in semi-natural conditions, using experimental infections allowing for testing during the acute and the chronic phases. For this experiment, we used *Plasmodium relictum*, the most common avian malaria parasite in our study population [38, 39] and in most parts of Europe (MalAvi database; [40]), and its natural vector *Culex pipiens*.

## Methods

The experiment was carried out during the last 2 months of summer 2020. Twenty canaries (*Serinus canaria*, 10 females and 10 males, with age from a few months to 3 years as estimated from birth years indicated on their rings) were placed into external aviaries (4.6 m × 4.6 m, 2.7 m high) overlaid with mosquito-proof netting and enriched with numerous perches, 7 days before their first exposure to mosquitoes. We provided them with canary food mix, apple and chicory, sand, and water for drinking and to bathing.

The *C. pipiens pipiens* mosquito population was initiated in September 2017 with wild clutches collected in the Dorigny forest, on the campus of the University of Lausanne, Switzerland (46.522565, 6.577927), and reared using standard protocols [41]. New wild individuals were mixed into the lab population in August 2018. Mosquitoes were hosted in a room with a constant temperature of 25 °C and 60% humidity, with a photoperiod lasting from 6:30 am to 9:30 pm, to match the external photoperiod.

The *P. relictum* strain (lineage SGS1) used in this experiment was isolated from a house sparrow (*Passer domesticus*) in January 2019 in the Dorigny forest and injected into a canary. The *Plasmodium* strain was maintained through regular passages across our stock canaries (15 times) using intraperitoneal (i.p.) injections [31]—except for the ninth passage, which was made by mosquitoes—until the beginning of the experiment. Experimental infection was performed by i.p. injection of 150 µL of a 1:1 mix of blood from three infected birds and 1X phosphate-buffered saline (PBS).

After the pre-infection exposure (see below), 12 experimental canaries (six males and six females) were infected as described above. We infected more than half of the birds to anticipate the probability that some of them would not catch the infection. The eight other experimental birds (four males and four females) were similarly injected with 150 µl of a 1:1 blood and PBS mix, except that the blood originated from three uninfected birds. The birds were blood-sampled by medial metatarsal venipuncture 6 days post-inoculation to check their infection status using a nested polymerase chain reaction (PCR) protocol targeting *Plasmodium cytb* [42].

### Experimental procedure

The 20 birds were exposed to mosquitoes three times: pre-infection (8 days before infection), 12 days post-infection (during acute phase) and 40 days post-infection (during chronic phase, [14]). Pre-infection exposure was considered as a control to account for individual variations in vertebrate hosts’ attractiveness.

Female mosquitoes aged between 5 and 30 days after emergence were placed inside the aviaries in rearing cages 30 h before the start of the experiment for acclimation. Sugar in their cages was replaced with water 24 h before and removed 6 h before the start of the exposure. The experiments started at sunset when the mosquitoes were released in the aviaries (pre-infection exposure: *N* = 750 mosquitoes; acute phase exposure: *N* = 425; chronic phase exposure: *N* = 749). The number of mosquitoes released varied slightly between experiments due to the fluctuating reproductive success of our freshly installed mosquito lineage in the laboratory. They were left in the aviary with the birds for the entire night. Mosquitoes were collected the next morning using insect-catching nets and mouth and hand insect vacuums. Following the third exposure session (i.e. chronic phase), the proportion of blood-fed mosquitoes was low—likely because the temperatures were lower at the end of summer—so we allowed them to feed an additional night. The blood-fed mosquitoes (pre-infection: *N* = 568; acute: *N* = 228; chronic: *N* = 266) were killed by putting them in a −20 °C freezer. The third (chronic phase) exposure session was done with only 19 birds, because one uninfected female died 2 weeks after the second exposure session.

Blood from birds was sampled in both acute and chronic stages by medial metatarsal venipuncture at 9:30 am the morning following the exposure experiment nights, to measure their parasitaemia. We also prepared blood smears for microscopic examination of gametocytaemia and weighed the birds to the nearest 0.1 g using an electronic balance. Blood smears were fixed with pure methanol and stained with Giemsa 7.5% during 45 min. Gametocytaemia was then assessed by counting the number of mature gametocytes found in 10,000 red blood cells.

### Molecular analyses

DNA from birds’ blood and from mosquitoes’ blood meals was extracted by using the DNeasy Blood&Tissue Kit (Qiagen, Switzerland). Prior to the first exposure experiment, birds were genotyped using a microsatellite amplification protocol adapted from Melo and Hansson [43] (Additional file 1: Text S1 and Table S1). Blood-fed mosquitoes were dissected to remove the head and the thorax before the digestion part of the DNA extraction protocol (pre-infection: *N* = 292; acute: *N* = 228; chronic: *N* = 266). DNA from blood meals was then genotyped with the same protocol as birds in order to identify its origin. Bird parasitaemia was measured using a TaqMan quantitative PCR protocol adapted from Christe et al. [44] (Additional file 1: Text S2).

###  Genotyping

Amplified fragments were separated by capillary electrophoresis using a 3100 Genetic Analyzer® (Applied Biosystems). Genotypes were evaluated on the GeneMapper® software v.4. Samples with more than two alleles in at least one of the markers, which could be caused by mixed blood meals (mosquito feeding on two or more birds) or contamination during lab procedures, were discarded (68/786 mosquitoes). Identity analyses were performed with Cervus© software v. 3.0.7 [45], with the minimum number of matching loci set at four and two fuzzy matches allowed. We identified 687 blood meals over the 786 tested mosquitoes.

### Statistical analyses

One female bird injected with infected blood did not develop the infection. Since the molecular diagnostics were performed at the end of the experiment, this female remained in the experiment but was removed from the statistical analyses. Thus, the final number of mosquitoes included in the analyses was 237 for the first exposure session, 194 for the second and 230 for the third. For the sake of simplicity, here we define one bird’s “attractiveness” as the number of mosquitoes found with its blood in their abdomens, although factors other than pure attractiveness could influence biting. First, we evaluated individual attractiveness during the three exposure sessions as a function of bird identity in a generalized linear model with a quasi-Poisson error distribution to assess whether birds differed in attractiveness. The significance of the explanatory variable was assessed by *F*-test comparing the deviance between models with and without the variable. Then, we calculated the individual change of attractiveness between the second exposure session (acute phase) and the first exposure session (pre-infection) as $\frac{{n}_{ind\_session\_2}}{194}-\frac{{n}_{ind\_session\_1}}{237}$, and $\frac{{n}_{ind\_session\_3}}{230}-\frac{{n}_{ind\_session\_1}}{237}$ for the third exposure session (chronic phase), *n*_ind_ being the number of mosquitoes found with the focal bird blood in its abdomen. We used Wilcoxon rank sum tests to compare infected and control birds’ change in attractiveness. We also compared the attractiveness of infected and control birds within each exposure session using Wilcoxon rank sum tests. We also used Wilcoxon rank sum tests to evaluate the effect of bird sex on attractiveness. Finally, we evaluated the link between attractiveness and age, as well as infection intensity (parasitaemia and gametocytaemia) in the acute and chronic phases using Spearman’s rank correlation. Statistical analyses were performed on R v4.2 [46] on RStudio v1.3.1056.

## Results

The average bird’s parasitaemia was 0.753 ± 0.614 and 0.006 ± 0.012 in the acute and chronic stages of infection, respectively. Gametocytes were present in all infected birds during their acute phase (mean number of gametocytes per 10,000 red blood cells ± SD: 52.91 ± 35.41, range: 3–118) but were detected on only two blood smears during the chronic phase (4 and 10, respectively). Gametocytaemia was correlated with parasitaemia during the acute phase (Spearman’s correlation: *r*_s_ = 0.8, *P* = 0. 0005). We recaptured 78% (*N* = 587), 68% (287) and 65% (490) of released mosquitoes after the first, second and third exposure sessions, respectively, among which 97% (568), 79% (228) and 54% (266) were blood-fed.

**Fig. 1:**
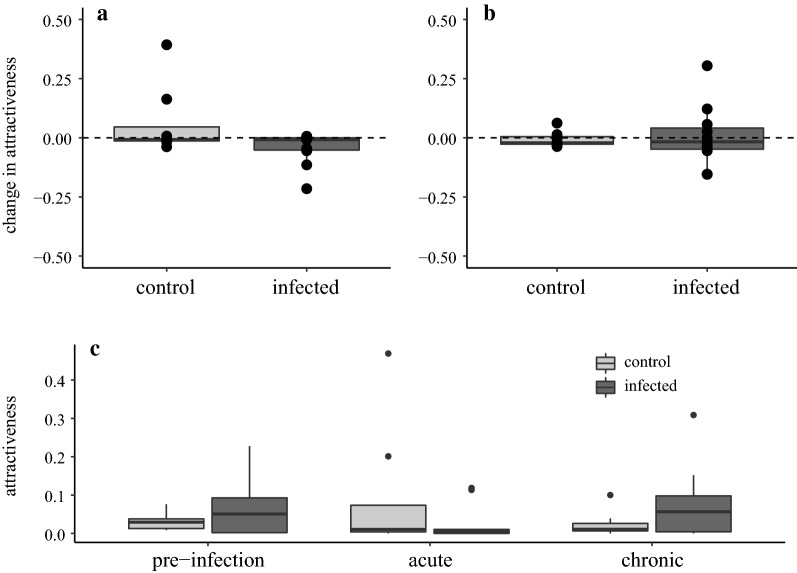
Changes in individual attractiveness between the first and **a** second (acute phase) and **b** third (chronic phase) exposure sessions, and **c** individual attractiveness during each exposure sessions. Here, “attractiveness” is defined as the number of total retrieved mosquitoes whose blood meal genotype matched the focal bird. We show proportions—instead of counts—as the total number of retrieved mosquitoes differed among exposure sessions

The mean number of mosquitoes per bird over the three sessions was 11.6 (95% CI: 7.4–17.1). Individual attractiveness was variable (*F* = 2.4, *P* = 0.01), with a few birds showing a large variance (e.g. one bird's blood was found in 18, 9 and 91 mosquitoes), while others had consistently low (e.g. 3, 0, 2) or high attractiveness (e.g. 30, 23, 35). Infected and control birds did not significantly differ in their change in attractiveness between the first and second exposure sessions (*W* = 60, *P* = 0.20, Fig. 1a) nor between the first and third exposure sessions (*W* = 41, *P* = 0.86, Fig. 1b). The number of bites received by the two treatment groups did not differ significantly within the first (*W* = 40, *P* = 0.77), second (*W* = 54, *P*-value = 0.42) or third (*W* = 29.5, *P* = 0.4381) exposure sessions (Fig. 1c). Male and female attractiveness did not differ significantly (first: *W* = 54, *P* = 0.49; second: *W* = 40.5, *P* = 0.74; and third session: *W* = 33, *P* = 0.56; all sessions together: *W* = 408.5, *P*-value = 0.96), and there was no interaction of bird sex with infection (Kruskal–Wallis acute: *χ*^2^_3_ = 3.41, *P* = 0.33; chronic: *χ*^2^_3_ = 1.21, *P* = 0.75).

Neither gametocytaemia (*r*_s_ = −0.01, *P* = 0.97) nor parasitaemia in the acute phase (*r*_s_ = 0.29, *P* = 0.40), nor parasitaemia in the chronic phase (*r*_s_ = 0.23, *P* = 0.53) correlated significantly with attractiveness. We did not analyse the effect of gametocytaemia during the chronic phase, as only two birds had gametocytes detectable on blood smears. Finally, there was a positive association between bird’s age and number of bites during the second exposure session (*r*_s_ = 0.55, *P* = 0.015) but not during the first (*r*_s_ = 0.03, *P* = 0.9) or the third (*r*_s_ = 0.17, *P* = 0.5) sessions.

## Discussion

In the present study, we tested in semi-natural conditions whether, as observed under laboratory conditions (e.g. [14, 23]), malaria infection in birds influenced the number of mosquito bites they received. Overall, neither infection status nor intensity significantly impacted birds’ probability of being bitten by mosquitoes. This suggests that, in conditions that are close to the wild environment, other factors play an antagonistic or a stronger role in influencing mosquito host detection, selection and biting than malaria-related increase in attractiveness. For instance, host metabolic rate (i.e. thermoregulation and CO_2_ emission) may have a different impact on mosquito behaviour according to experimental conditions. Carbon dioxide is used in long-range detection by mosquitoes [47], and it has been shown that malaria-infected birds may have lower CO_2_ production than uninfected individuals [48]. Whereas in laboratory experiments CO_2_ may not play a major role in host detection and selection, since hosts and mosquitoes are usually in close proximity (i.e. a few tens of centimetres), differences in CO_2_ emission could be preponderant in host detection when the distance between hosts and mosquitoes is greater. Further, spatial positioning of the birds in the environment (large external aviaries here) could influence their probability of encounter with mosquitoes [49], as suggested by the fact that we retrieved higher densities of mosquitoes in some corners of the aviary than others (personal observations).

Assuming avian malaria generally manipulates bird attractiveness, our experiment might have missed it due to unintentional artificial selection. Indeed, we used a *P. relictum* strain that had been passaged 15 times from birds to birds through intraperitoneal injections, except for the ninth passage which was done by mosquitoes. This represents a release from the selective pressure to attract mosquitoes, which might be enough for this strain to have lost its ability to alter birds’ attractiveness [29]. Alternatively, manipulation of birds’ attractiveness might be specific, and we missed it by injecting in canaries a strain originally isolated from a house sparrow; however, this explanation does not seem very likely, as *P. relictum* SGS1 is a very generalist and widespread lineage [40]. In addition, avian malaria is caused by a high diversity of *Plasmodium* species and lineages, which differ in morphology, specificity and vectors, and the ability to alter hosts’ attractiveness to mosquitoes might also vary strongly among lineages. Finally, the two main limitations of our study are the weak statistical power linked with a low sample size and, directly related to our experimental design, the non-independence of observations. Indeed, the level of individual attractiveness directly impacts the number of mosquitoes available to bite other birds. However, we introduced high numbers of mosquitoes into the cage to minimize this effect. During both the acute and chronic exposure session, an important proportion of mosquitoes remained unfed at the end of the experiment (21–46%).

Although not significant, we found however a trend in infected birds, which tended to receive fewer bites in the acute phase than before infection, which is congruent with results reported by Cornet et al. [14]. This might be explained by a lower production of some cues used by mosquitoes to detect their host (such as CO_2_, [48]) and/or by the lower nutritional value of acutely infected blood. Indeed, at the end of each erythrocytic cycle of replication, *Plasmodium* parasites rupture the red blood cell that they occupy. This replication is at its highest intensity during the acute phase of infection, which corresponds to a low density of red blood cells: haematocrit correlates negatively with parasitaemia ([50] but see [31]). Consequentially, as the main source of proteins for blood-feeding mosquitoes are the erythrocytes, a decrease in their density makes the blood less nutritious. Indeed, mosquitoes prefer hosts with higher haematocrit [14], although the exact mechanism by which mosquitoes evaluate blood quality is unclear. Avoidance of low-quality blood by mosquitoes during the acute phase might counterbalance a potential effect of *Plasmodium*-associated attractants.

Overall, our results question the adaptive nature of increased attraction found in infected birds in some laboratory experiments [14, 23]. Indeed, not only did these experiments take place in small, confined spaces, with mosquitoes being closely and rather equally exposed to both birds’ cues (e.g. odours, CO_2_), which is likely rare in the wild, but they also produced relatively small differences in attraction [14, 23]. In more natural conditions regarding spatial distribution of host cues, this difference in attractiveness might not suffice to result in more mosquito bites and thus to confer avian *Plasmodium* sp. a transmission advantage, as would be suggested by our results. Although evidence of enhanced attraction of malaria-infected hosts is more conclusive in mammals, mechanisms of manipulation might differ from those potentially existing in birds, notably due to the role of the uropygial gland in bird body odour [51] and other anatomical differences (e.g. nucleated red blood cells, feathers). In conclusion, while our study has some limitations, such as the possible bias induced by the inability to identify mixed blood meals [49], the weak statistical power and the inevitable non-independence of observations intrinsic to our experimental design, it highlights the importance of confronting the results obtained in the laboratory with those obtained under natural conditions, as evaluating the adaptive nature of an observed phenomenon is crucial [6].

## Supplementary Information

**Additional file 1: Text S1**. Genotyping method. **Table S1**. Primers used for amplification of microsatellite regions in canaries. Text S2. Parasitaemia measurement in bird blood.

**Additional file 2: Dataset S1**. Results from the three exposure sessions.

## Data Availability

The protocols and the data set are provided in supplementary material. Raw data (microsatellite and qPCR runs) are available on figshare: https://doi.org/10.6084/m9.figshare.19596646.v1.
