# Supplementary material for: Experiment in semi-natural conditions did not confirm the influence of malaria infection on bird attractiveness to mosquitoes
Source: Parasit Vectors. 2022 Jun 2;15:187. doi: 10.1186/s13071-022-05292-w (PMC9164852; doi:10.1186/s13071-022-05292-w)
Supplement: Supplementary file 1 — Additional file 1: Text S1. Genotyping method. Table S1. Primers used for amplification of microsatellite regions in canaries. Text S2. Parasitaemia measurement in bird blood. [file 13071_2022_5292_MOESM1_ESM.docx]

Experiment in semi-natural conditions did not confirm the influence of malaria infection on mosquito biting preference observed in the lab.

**Camille-Sophie Cozzarolo*^1^, Romain Pigeault^1,2^, Julie Isaïa^1^, Jérôme Wassef^1^, Molly Baur^1^, Olivier Glaizot^1,3^† and Philippe Christe^1^†**

Additional file 1

# Text S1. Genotyping method.

One or two microsatellite loci were amplified by reaction (Table S1). Reactions were performed in a volume of 10µL composed of 5.2 µL of RNase-Free water (or 5.6 in reactions containing only one pair of primers), 3µL of QIAGEN Multiplex PCR Master Mix, 0.2 µL of each primer (final concentration: 0.2mM) and 1µL of DNA. Cycling conditions were the following: 15min of initial denaturation at 95°C, then 40 cycles composed of a 30sec denaturation phase at 94°C, a 1min30 annealing phase and a 1min elongation at 72°C, and finally 30min of final extension at 60°C (adapted from [1]).

# Table S1 Primers used for amplification of microsatellite regions in canaries.

| Mix | Locus | Sequence | Fluorescence dye | Annealing temperature |
| --- | --- | --- | --- | --- |
| mix A | Cuμ4 | F: AATTGCATAAATGTGATCCAC  R: AAATGAAATGTGGTAGAATTCC | FAM | 60°C |
| mix B | LOX7 | F: AACCTAAGCACATTTATTCAGC  R: AACAAATAACATAGGTCAGAAGC | ATTO532 | 55°C |
|  | Lswμ18 | F: TTGCTGAAAGAAGTACTAAGA  R: CTGKTTGCAGGATATGTATAC | ATTO550 |  |
| mix C | LOX1 | F: ATGATGGTAAGTCTAATGAAAGC  R: CCACACACATTCACTCTATTG | FAM | 54°C |
| mix D | Cuμ28 | F: GAGGCACAGAAATGTGAATT  R: TAAGTAGAAGGACTTGATGGCT | ATTO550 | 60°C |
|  | LOX3 | F: TTCTGTGGTGAAGTTTTCTGGAG  R: CCAACCCATTCCATGACAAC | FAM |  |

# Text S2. Measurement of parasitaemia in bird blood

A fragment of bird *18S rRNA* and *Plasmodium* sp. *cytb* genes were amplified using primers and probes designed by [2], with the only difference that the *Plasmodium* *cytb* probe was labelled with the Texas Red reporter instead of CY3. Parasite and host fragments were amplified in separate reactions, in triplicates, using BioRad CFX96™ Real-Time PCR Detection System. Final volume per reaction was 20µL, including 10µL of SSo Advanced BioRad Universal Probe Mix, 0.3µM of each primer, 0.5ng/µL of DNA and volume was adjusted with water; parasite and host reactions differ in the concentration of probes, with 0.8µM of parasite probe and 0.2µM of host probe. Cycling conditions started with 2min at 50°C, followed by 10min at 95°C, and then 48 cycles with the following parameters: a) parasite reactions: 15sec at 95°C, 30sec at 50°C and 30sec at 60°C; b) host reactions: 15sec at 95°C and 1min at 59.2°C. Parasitaemia was calculated as $\frac{{10}^{\frac{Ct_{parasite}-I_{parasite}}{m_{parasite}}}}{{10}^{\frac{Ct_{host}-I_{host}}{m_{host}}}}$ , Ct being the mean of the three measured Ct, I the intercept of the standard curve and m the slope of the standard curve, as in [3]. Parasitaemia was log-transformed in order to normalize the distribution.

# References

1. Melo M, Hansson B. Identification of 15 polymorphic microsatellite loci in the Príncipe seedeater (*Serinus rufobrunneus*) and assessment of their utility in nine other *Serinus* species (Fringillidae, Aves). Mol Ecol Notes. 2006;6:1266–8.

2. Christe P, Glaizot O, Strepparava N, Devevey G, Fumagalli L. Twofold cost of reproduction: an increase in parental effort leads to higher malarial parasitaemia and to a decrease in resistance to oxidative stress. Proc R Soc B Biol Sci. 2012;279:1142–9.

3. Cozzarolo C-S, Jenkins T, Toews DPL, Brelsford A, Christe P. Prevalence and diversity of haemosporidian parasites in the yellow-rumped warbler hybrid zone. Ecol Evol. 2018;1–14.
